# Boiled, sprouted, and raw cowpea‐incorporated diets modulate high‐fat diet‐induced hypercholesterolemia in rats

**DOI:** 10.1002/fsn3.727

**Published:** 2018-08-13

**Authors:** Ruvini Liyanage, Oshini Perera, Gusthingna W. A. S. Lakmini, Pabodha Weththasinghe, Rizliya Visvanathan, Chathuni Jayathilake, Barana C. Jayawardana, Janak Vidanarachchi, Ramiah Sivakanesan

**Affiliations:** ^1^ Division of Nutritional Biochemistry National Institute of Fundamental Studies Kandy Sri Lanka; ^2^ Postgraduate Institute of Agriculture University of Peradeniya Peradeniya Sri Lanka; ^3^ Department of Agricultural Systems Faculty of Agriculture Rajarata University of Sri Lanka Anuradhapura Sri Lanka; ^4^ Department of Animal Science Faculty of Agriculture University of Peradeniya Peradeniya Sri Lanka; ^5^ Department of Biochemistry Faculty of Medicine University of Peradeniya Peradeniya Sri Lanka

**Keywords:** cowpea, hypocholesterolemia, serum antioxidants, serum lipids, Wistar rats

## Abstract

This study was carried out to investigate the effect of processed (boiled and sprouted) cowpea‐incorporated experimental diets on serum cholesterol and serum antioxidant capacity in high‐fat diet (HFD)‐fed Wistar rats. Seven weeks old male Wistar rats were fed 20% fat as a control (CD), for comparison with 20% fat‐enriched diets containing 20% whole raw cowpea diets (Bombay Raw Diet; BRD and MI35 Raw Diet; MRD), boiled cowpea diets (Bombay Boiled Diet; BBD and MI35 Boiled Diet; MBD) and sprouted cowpea diet (Bombay Sprouted Diet; BSD) for 6 weeks. The increase in serum total cholesterol as a result of high‐fat diet was significantly countered by boiled and raw cowpea‐incorporated diet‐fed rats. Increased serum non‐HDL‐C level caused by HFD was significantly (*p *<* *0.05) countered by raw, boiled, and sprouted cowpeas, while HDL–C was increased by raw MI and boiled Bombay incorporated diets. Boiling has improved the hypocholesterolemic ability of Bombay cowpea and BBD has significantly (*p *<* *0.05) modulated serum HDL‐C level and liver weight in rats. These findings were supported significantly high soluble fiber content in processed cowpea powder than that in raw cowpea powder. The decrease in serum antioxidant activity as a result of HFD was significantly countered by BRD. Processing has reduced the antioxidant activity in cowpeas and serum antioxidant activity in rats. Cecal lactobacilli population was significantly high in all cowpea diet‐fed groups compared to control. Modulated serum cholesterol level in cowpea diet‐fed rats was accompanied by dietary fiber composition, antioxidant activity in cowpeas and fecal weight, cecal weight and cecal lactobacilli population in rats compared to control. Both processed and raw cowpea‐incorporated diets have modulated HFD‐induced hypercholesterolemia by modulating serum antioxidative capacity, cholesterol metabolism, and cecal fermentation.

## 1 INTRODUCTION

Legume consumption is considerably high in developing countries and is used as a main source of protein compared to the developed world. With the shift in dietary pattern, throughout the world legume consumption has reduced dramatically during the past few decades. Many studies have revealed a clear relationship between diet and noncommunicable diseases. International Diabetes mellitus guidelines recommended the consumption of legumes for controlling of the glycemic index. Some epidemiological studies have strongly indicated that legume consumption reduces the risk of coronary heart diseases (Bazzano, Thompson, Tees, Nguyen, & Winham, 2011; Ha et al., 2014). From previous studies, hypocholesterolemic ability of 7s globulin from cowpeas, cowpea protein isolates, and cowpea seeds was well documented (Ferreira et al., 2015; Frota, dos Santos Filho, Ribeiro, & Arêas, 2015; Frota, Mendonça, Saldiva, Cruz, & Arêas, 2008). From our previous study, we observed that raw cowpea‐incorporated experimental diets modulated serum lipids and serum antioxidant activity in Wistar rats (Perera et al., 2016; Weththasinghe, Liyanage, Vidanarachchi, Perera, & Jayawardana, 2014).

Legumes contain a range of nutrients and bioactive components and the fatty acid profile, dietary fiber, bioactive peptides and favorable amino acids, isoflavones and antioxidants may contribute to the functional properties of legumes (Liyanage et al., 2014; Pastor‐Cavada, Juan, Pastor, Alaiz, & Vioque, 2009; Rochfort & Panozzo, 2007). However, some of these bioactive components are considered as antinutritive factors which act to reduce nutrient intake, digestion, absorption, and utilization; they include tannins, saponins, phytates, flavonoids, alkaloids, cyanogenic, glycosides, and trypsin (Aremu, Olaofe, Basu, Abdulazeez, & Acharya, 2010; Audu, Aremu, & Lajide, 2013; Copeland & McDonald, 2001).

Legumes are generally consumed after various processes and with processing, the antinutritional factors are improved. Germination is one of the most common techniques used to reduce most of the antinutritional factors in legumes (Alonso, Orúe, & Marzo, 1998). It has been shown that cooking modulates nutritional and biochemical parameters of the legumes (Nergiz & Gökgöz, 2007; Nisha, Singhal, & Pandit, 2005; Ramesh & Tharanathan, 1999). However, there has been very little research reported on the effect of processing on functional properties of legumes (Dahiya et al., 2015). Hence in this study, effect of processed cowpea‐incorporated experimental diets on in vivo hypocholesterolemic effect and serum antioxidant activity were studied in an animal experimental model. This will provide useful information to the industrialists and others alike on the subsequent incorporation of the studied samples into food products by producing natural, cheap, and adaptable functional foods.

## 2 MATERIALS AND METHODS

### 2.1 Preparation of cowpea powder

#### 2.1.1 Preparation of raw cowpea powder

Cowpea cultivars “Bombay”, and “MI35” were purchased from government seeds farm Palwehera, Dambulla, Sri Lanka. Dry cowpea seeds were visually inspected and defective seeds were discarded before the preparation of powder. Cowpea seeds were washed and oven dried in drying oven (YAMATO IC600, Yamato Scientific Co., Ltd., Japan) at 60°C until a constant weight was obtained and ground using a grinder (MX‐151SG1, Panasonic Co., Ltd, China) to a fine consistency.

#### 2.1.2 Preparation of boiled cowpea powder

Cowpea seeds were washed and soaked overnight in distilled water in room temperature and boiled for 30 min in distilled water. Boiled seeds were then air dried for 24 hr and oven dried in drying oven (YAMATO IC600, Yamato Scientific Co., Ltd., Japan) at 60°C until a constant weight was obtained and ground using a grinder (MX‐151SG1, Panasonic Co., Ltd, China) to a fine consistency.

#### 2.1.3 Preparation of sprouted cowpea powder

Cowpea seeds were soaked in distilled water overnight and allowed for germination on wet kitchen towels in room temperature for 2 days. They were then air dried for 24 hr and oven dried using YAMATO IC600 drying oven (Yamato Scientific Co., Ltd., Japan) at 60°C until a constant weight was obtained and ground using a grinder (MX‐151SG1, Panasonic Co., Ltd, China) to a fine consistency.

### 2.2 Animals and diets

Male Wistar rats (7 weeks old) were purchased from the Medical Research Institute, Colombo, Sri Lanka. They were housed individually in cages with free access to food and water. The animal facility was maintained on a 12 light/dark cycle at a temperature of 23 ± 1°C and relative humidity of 60 ± 5%. The rats were randomly assigned into five groups (*n *=* *5). There were no significant (*p *<* *0.05) differences in body weights and serum total cholesterol concentrations among groups at the beginning of the experiment. Proximate composition of the cowpea cultivars in raw and processed forms was determined by the procedure of the Association of Official Analytical Chemists (AOAC, 1990). The insoluble dietary fiber (IDF), soluble dietary fiber (SDF), and total dietary fiber (TDF) content of the cowpea powder were analyzed using a combination of enzymatic and gravimetric procedures of Prosky, Asp, Schweizer, DeVries, and Furda (1988). Antioxidant activity and phenol content of cowpea powders were measured. Antioxidant activity was measured by FRAP method (Al‐Farsi, Alasalvar, Morris, Baron, & Shahidi, 2005) and phenol content was measured by Folin‐Ciocalteu assay (Singleton & Rossi, 1965). Experimental diets were prepared according to AIN 93G semipurified rodent diets (Table 1). The experimental rats were fed for 6 weeks, with 20% fat as a basal diet (HFD), for comparison with 20% fat‐enriched diets containing 20% whole raw Bombay and MI35 cowpea powder (BRD and MRD), boiled Bombay and MI35 cowpea powder (BBD and MBD) and sprouted Bombay cowpea powder (BSD). There was no significant difference (*p *<* *0.05) in food intake and final body weight among groups at the end of the experimental period. The blood samples (1 ml) were taken at the beginning and at the end of the 6 weeks between 09.00 and 10.00 in the morning from the jugular vein of fasting rats after anesthetizing. The samples were taken into tubes without any anticoagulant. After the samples were allowed to stand at room temperature for 2 hr, the serum was separated by centrifugation at 1,500 × *g* for 20 min. At the end of the 6‐week experimental period, all feces excreted during last 3 days were collected. The rats were anesthetized and killed, and the livers and cecum were quickly removed, washed with cold saline (9 g NaCl/L), blotted dry on filter paper, and weighed before freezing for storage. This experimental design was approved by the Animal Experiment Committee of Faculty of Veterinary Medicine and Animal Science, University of Peradeniya, Sri Lanka. All animal procedures conformed to standard principles described in *Guide for the Care and Use of Laboratory Animals* (The National Research Council, 2011).

**Table 1:** Crude fat, crude fiber, crude protein, ash, carbohydrate (CHO), and dry matter content (dry g/100 g) of cowpea powder

Cultivar	Crude fat	Crude protein	Crude fiber	Ash	CHO	Dry matter	Dietary fiber		
							Insoluble	Soluble	Total
Bombay raw	3.45 ± 0.06	23.45 ± 0.62	6.85 ± 0.07	3.55 ± 0.04	58.89 ± 0.44	96.19 ± 0.06	19.12 ± 0.22^b^	0.09 ± 0.01^c^	19.18 ± 0.18^b^
Bombay boiled	3.08 ± 0.12	25.40 ± 0.34	6.38 ± 0.03	4.80 ± 0.04	56.50 ± 0.66	96.34 ± 0.03	10.93 ± 0.01^c^	0.33 ± 0.02^b^	11.26 ± 0.09^c^
Bombay sprouted	3.44 ± 0.13	24.06 ± 0.16	6.24 ± 0.12	3.21 ± 0.18	59.29 ± 0.36	95.91 ± 0.21	10.24 ± 0.13^c^	0.32 ± 0.03^b^	10.56 ± 0.13^c^
MI 35 raw	3.24 ± 0.05	23.45 ± 0.01	6.49 ± 0.17	3.90 ± 0.04	59.63 ± 0.21	96.71 ± 0.06	26.99 ± 0.25^a^	0.11 ± 0.03^c^	27.10 ± 0.15^a^
MI 35 boiled	3.11 ± 0.08	24.06 ± 0.07	6.24 ± 0.04	3.21 ± 0.28	59.29 ± 0.41	95.91 ± 0.33	18.15 ± 0.13^b^	0.49 ± 0.07^a^	18.64 ± 0.13^b^

*Note*

Values are expressed as means ± *SD*. Values in each column with different superscripts are significantly different (*p* < 0.05).

### 2.3 Serum lipid analysis

Total cholesterol (TC) and HDL‐cholesterol (HDL‐C) concentrations in the serum were determined enzymatically using commercially available reagent kits (ProDia Internationals, Germany). The non‐HDL‐cholesterol (non‐HDL‐C) concentration was calculated as follows: [non‐HDL‐C] = [TC]‐[HDL‐C].

### 2.4 Serum antioxidant activity (AOA) analysis

Serum antioxidant activity was measured by Ferric Reducing Antioxidant Power Assay (FRAP). Working FRAP solution was prepared by mixing Acetate buffer, 300 mM, 10 mM TPTZ (2, 4, 6, Tripyridyl‐s‐Tartazine) in 40 mM HCl and FeCl_3_ 20 mM in 10:1:1 ratio. An amount of 20 μl of separated serum was reacted with 1 ml of working FRAP. The absorbance was measured at 593 nm (UV‐VIS‐2460, Shimadzu, Kyoto, Japan) exactly after 4 min and antioxidant activity was analyzed by relating to 1 mM Ferrous sulfate standard (Al‐Farsi et al., 2005).

$$\begin{array}{cc} & \text{FRAP value}\;(\mu \text{mol}/L)=\\ & (A_{\text{sample}}/A_{\text{standard}})*\text{Concentration of working FRAP} \end{array}$$

Where,

A_sample_ is the absorbance of the sample,

A_standard_ is the absorbance of standard and

Concentration of working FRAP was 1,000 μmol/L.

### 2.5 Growth of bacteria in the cecum

Anaerobes, lactobacillus, and coliform from the cecum were inoculated and incubated on Wilkins Chalgren anaerobe agar (Oxoid Ltd, England), lactobacillus MRS agar (Oxoid Ltd, England), and MacConkey agar (Himedia, India) at 37°C as previously described (Liyanage et al., 2007, 2009).

### 2.6 Statistical analysis

Data are presented as the mean and standard deviation for serum TC, HDL‐C and, non‐HDL‐Cat the prescribed times. The significance of difference among treated groups was determined by ANOVA with Duncan's multiple range test using SAS statistical software (SAS Institute, Cary, NC, USA).

## 3 RESULTS AND DISCUSSION

### 3.1 Dietary fiber content, antioxidant activity, and phenol content in cowpea powder

Soluble dietary fiber content was significantly high (*p *<* *0.05) in processed cowpea powder than that in raw cowpea powder, while insoluble dietary fiber content was significantly low (*p *<* *0.05) in processed cowpea powder (Table 1). Total dietary fiber content in raw MI35 was significantly higher (*p *<* *0.05) than MI35 boiled, Bombay raw and Bombay sprouted powder. In this study, boiling and sprouting have improved the soluble dietary fiber content in cowpea compared to raw cowpea and these findings were supported by findings of our study on mung beans (Liyanage et al., 2018). Antioxidant activity (AOA) was significantly lower (*p *<* *0.05) in processed cowpea powders than the raw cowpea powder (Table 2). Phenol content was significantly lower (*p *<* *0.05) in boiled cowpea powders than that in raw cowpea powders. These findings were supported by previous findings showing that boiling reduced the total phenol content and antioxidant actvity in mung beans (Liyanage et al., 2018). Phenol content (Table 2) in sprouted Bombay cowpea powder was not significantly different (*p *<* *0.05) compared to that in raw Bombay cowpea powder (Table 3).

**Table 2:** Antioxidant activity (AOA) and total phenolic content (TPC) of cold and hot extracts of cowpea powder

Cultivar	Cold extract			Hot extract		
	AOA FRAP (μmol/g dry powder)	AOA DPPH (IC_50_ in mg)	TPC (mg/g dry powder)	AOA FRAP (μmol/g dry powder)	AOA DPPH (IC_50_ in mg)	TPC (mg/g dry powder)
Bombay raw	18.21 ± 1.68^a,Q^	422.72 ± 2.68^e,S^	4.62 ± 0.04^a^	24.07 ± 1.39^a,R^	255.70 ± 0.38^e,T^	5.57 ± 0.35^a^
Bombay boiled	5.61 ± 0.54^c,Q^	780.23 ± 0.68^a,S^	3.17 ± 0.1^c^	7.86 ± 0.68^c,R^	680.22 ± 0.88^a,T^	3.25 ± 0.65^b^
Bombay sprouted	5.23 ± 0.21^c,Q^	720.72 ± 2.56^b,S^	4.29 ± 0.05^b^	8.21 ± 1.02^c,R^	601.25 ± 0.40^b,T^	5.01 ± 1.38^a^
MI 35 raw	8.02 ± 0.04^b,Q^	586.63 ± 0.48^d,S^	2.63 ± 0.01^d^	10.94 ± 0.41^b,R^	347.90 ± 1.18^d,T^	2.70 ± 0.05^c^
MI 35 boiled	2.94 ± 0.01^d^	602.42 ± 2.45^c,S^	1.12 ± 0.04^e^	3.51 ± 0.02^d^	531.05 ± 1.08^c,T^	2.03 ± 0.28^d^

*Note*

Values are expressed as means ± *SD*. Values in each column with different superscripts a to d are significantly different (*p *<* *0.05). Values in each row with different superscripts Q to R for FRAP and S to T for DPPH for cold and hot extracts are significantly different (*p *<* *0.05).

**Table 3:** Composition of experimental diets (1 Kg)

Ingredients	Bombay (BRD)	Bombay Boiled (BBD)	Bombay Sprouted (BSD)	MI 35 (MRD)	MI 35 Boiled (MBD)	Control (CD)
Casein	153	149	148	153	152	200
Cowpea powder	200	200	200	200	200	–
Lard	293	293.8	293	293.5	293.7	300
Mineral Mixture^a^	35	35	35	35	35	35
Vitamin Mixture^a^	10	10	10	10	10	10
Cellulose Powder	40	40	40	40	40	40
Sucrose	100	100	100	100	100	100
L‐ Cystine	3	3	3	3	3	3
Choline	2.5	2.5	2.5	2.5	2.5	2.5
TBHQ	0.014	0.014	0.014	0.014	0.014	0.014
α‐ Corn Starch	163.49	166.69	168.49	162.99	163.79	309.49

*Note*

a AIN93G mineral and vitamin mixture.

### 3.2 Serum cholesterol level in rats fed experimental diets

**Figure 1:**
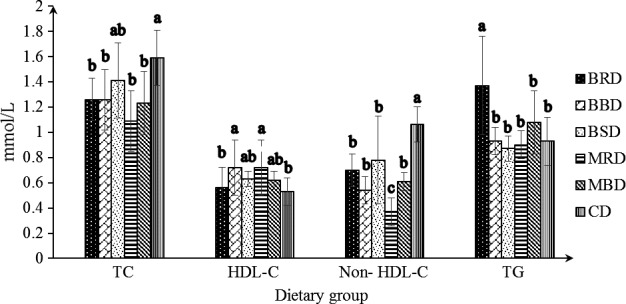
Serum Total cholesterol, High‐Density Lipoprotein cholesterol (HDL‐C), Non‐HDL‐cholesterol (Non‐HDL‐C), and triglyceride (TG) concentration in rats fed experimental diets for 6 weeks. Values are expressed as mean ± *SD*. Mean values within a group with different superscript letters are significantly different at *p *<* *0.05

Figure 1 shows the serum Total Cholesterol (TC), High‐Density Lipoprotein Cholesterol (HDL‐C), non‐High‐Density Lipoprotein Cholesterol (non‐HDL‐C), and serum antioxidant activity in rats fed experimental diets for 6 weeks. The serum TC level was significantly lower (*p *<* *0.05) in rats fed BRD, BBD, MRD, and MBD diets than that in the control, showing that both boiled and raw cowpea powder‐incorporated diets significantly reduced the HFD induced serum TC level in Wistar rats. The serum non‐HDL‐C level was significantly (*p *<* *0.05) lower in all cowpea‐incorporated diet‐fed rats compared to control. Serum HDL‐C level was significantly high (*p *<* *0.05) in BBD, MRD‐fed group compared to that in BRD and CD‐fed groups. Dietary fibers exert hypocholesterolemic effects by increasing fecal excretion of steroids (Sembries, Dongowski, Mehrländer, Will, & Dietrich, 2004). The Higher soluble fiber content in processed cowpeas may have favorably modulated serum non‐HDL‐C level in rats as shown previously (Pande, Platel, & Srinivasan, 2012; Rideout, Harding, Jones, & Fan, 2008). The impact of various soluble fibers on lipid metabolism has been well established both in humans and animal models (Aller et al., 2004; Levrat, Texier, Régerat, Demigné, & Rémésy, 1993). The results were in good accordance with the study on whole cowpea seeds and its protein isolates (Frota et al., 2008, 2015). Legumes are also good sources of saponins and phytosterols which may assist in decreasing the absorption of cholesterol from the gut. Clinical studies have suggested that saponins in legumes protect the human body from cancers and high blood cholesterol (Shi et al., 2004). Boiling has improved the hypocholesterolemic ability of Bombay cowpea and BBD has significantly (*p *<* *0.05) modulated serum HDL‐C level and liver weight (Table 4) in rats than those in raw Bombay cowpea diet‐fed rats. Significantly low TC level (*p *<* *0.05) in rats fed both raw and boiled cowpea‐incorporated diets was supported by significantly lower serum (*p *<* *0.05) non‐HDL‐C level, significantly higher fecal weight (Table 4) and significantly high dietary fiber content in cowpeas as shown previously (Bazzano et al., 2011).

**Table 4:** Initial body weight, final body weight, body weight gain, fecal weight, and cecal weight of rats fed experimental diets for 6 weeks

	Body weight			Liver weight (Wet/100 g/body weight	Fecal weight (g)	Cecal weight (g/100 g of BW)
	Initial (g/rat)	Final (g/rat)	Gain (g/rat/6 weeks)			
BRD	207.40 ± 16.45^a^	351.80 ± 20.14^a^	144.40 ± 9.01^a^	2.85 ± 0.15^b^	2.02 ± 0.44^ab^	0.64 ± 0.12^a^
BBD	209.20 ± 19.57^a^	333.40 ± 43.36^a^	124.20 ± 27.04^a^	2.58 ± 0.07^c^	2.22 ± 0.78^a^	0.68 ± 0.12^a^
BSD	209.60 ± 17.75^a^	335.60 ± 28.97^a^	126.00 ± 30.92^a^	2.82 ± 0.30^b^	2.20 ± 0.26^a^	0.65 ± 0.08^a^
MRD	215.20 ± 15.22^a^	345.00 ± 15.57^a^	129.80 ± 9.28^a^	2.91 ± 0.10^ab^	2.45 ± 0.33^a^	0.68 ± 0.11^a^
MBD	209.40 ± 18.75^a^	330.80 ± 24.48^a^	121.40 ± 10.80^a^	2.60 ± 0.19^c^	2.25 ± 0.78^a^	0.67 ± 0.21^a^
CD	222.60 ± 13.52^a^	351.80 ± 4.50^a^	128.00 ± 5.56^a^	3.11 ± 0.12^a^	1.62 ± 0.23^b^	0.46 ± 0.32^b^

*Note*

BRD = HFD + 20% Bombay raw cowpea powder, BBD = HFD + 20% Bombay boiled cowpea powder, BSD = HFD + 20% Bombay sprouted cowpea powder, MRD = HFD + 20% MI 35 raw cowpea powder, MBD = HFD + 20% MI 35 boiled cowpea powder CD = HFD + Casein powder. Values are expressed as means ± *SD*. Mean values within a column with different superscript letters were significantly different (*p *<* *0.05).

### 3.3 Serum antioxidant activity in rats fed experimental diets

**Figure 2:**
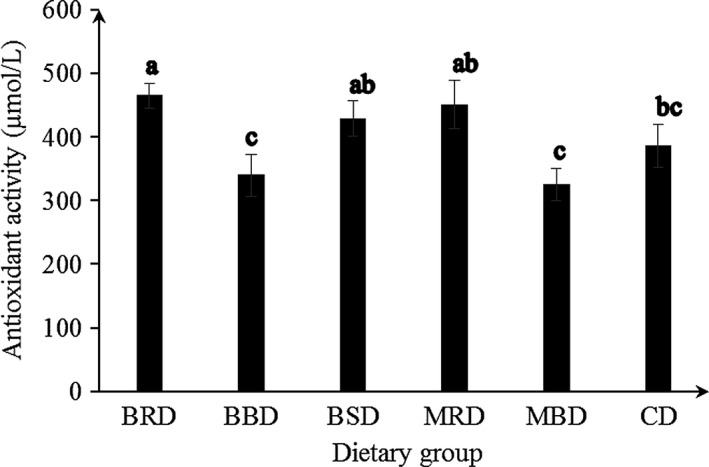
Serum antioxidant activity in rats fed experimental diets for 6 weeks. Values are expressed as mean ± *SD*. Mean values within a group with different superscript letters are significantly different at *p *<* *0.05

Serum antioxidant activity was significantly high (*p *<* *0.05) in BRD‐fed group compared to that in BBD, MBD, and CD (Figure 2). Significantly low (*p *<* *0.05) serum TC and non‐HDL‐C level in BRD‐fed rats was supported by significantly higher (*p *<* *0.05) serum antioxidant activity compared to control diet, and this was in agreement with previous studies showing that significantly lower serum lipid level may be due to higher antioxidant activities preventing lipid peroxidation (Jemai et al., 2008). However, the BBD and MBD‐fed rats had significantly (*p *<* *0.05) lower serum antioxidant activity compared to that in BRD and MRD‐fed rats and these data were accompanied by significantly low (*p *<* *0.05) antioxidant activity and phenol content in boiled Bombay cowpea powder (BB) and boiled MI35 powder than raw cowpeas. Thus, processing has reduced the antioxidant activity in cowpeas and serum antioxidant activity in rats.

### 3.4 Cecal fermentation in rats fed experiental diets

**Figure 3:**
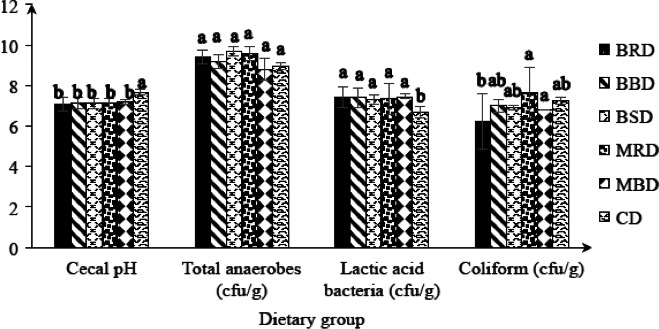
Cecal pH and cecal bacterial population of rats fed experimental diets for 6 weeks. Values are expressed as mean ± *SD*. Mean values within a group with different superscript letters are significantly different at *p *<* *0.05

There was no significant (*p *<* *0.05) difference in anaerobe bacterial population among groups (data not shown). Cecal *Lactobacillus* population (Figure 3) was significantly high (*p *<* *0.05) in all cowpea diet‐fed groups compared to control group followed by significantly lower (*p *<* *0.05) cecal pH and significantly higher ceceal weight (Table 4) in all the cowpea‐incorporated diet‐fed groups than the control group. The coliform population (Figure 2) was significantly lower (*p *<* *0.05) in BRD‐fed group than that in MRD or MBD‐fed groups. The reason for significantly higher cecal *Lactobacillus* population and significantly higher cecal weight in cowpea‐fed groups remains unclear. Undigested cowpea proteins or dietary fiber could have been used as substrates by *Lactobacillus* and have increased their population. Legumes do contain galactooligosaccharides (GOS), small unabsorbed carbohydrates that are rapidly fermented by the gut bacteria (Biesiekierski et al., 2011). Higher *Lactobacillus* population may have produced high propionic acid concentration and modulated serum cholesterol in rats fed cowpea‐incorporated diet as shown previously (Jenkins, Kendall, & Vuksan, 2000).

## 4 CONCLUSION

The increase in TC and non‐HDL‐C level as a result of HFD was significatly encountered by boiled, raw cowpea diets and boiled, sprouted, raw cowpea‐incorporated diets, respectively. Lower serum cholesterol level in cowpea‐fed rats was supported by higher serum antioxidant activity and higher cecal lactobacilli population compared to control diet. Both processed and raw cowpea‐incorporated diets have modulated HFD‐induced hypercholesterolemia by modulating serum antioxidative capacity, cholesterol metabolism, and cecal fermentation. Thus, boiled, sprouted, and raw cowpea may be effective in the treatment of hypercholesterolemia in humans and further studies in human volunteers need to be done to substantiate these findings.

## ETHICAL STATEMENT

The authors declare that there is no conflict of interest regarding the publication of this article. Protocols and procedures in this study were ethically reviewed and approved by the Animal Experiment Committee of Faculty of Veterinary Medicine and Animal Science, University of Peradeniya, Sri Lanka.
